# Stationary log-normal distribution of weights stems from spontaneous ordering in adaptive node networks

**DOI:** 10.1038/s41598-018-31523-1

**Published:** 2018-08-30

**Authors:** Herut Uzan, Shira Sardi, Amir Goldental, Roni Vardi, Ido Kanter

**Affiliations:** 1 Department of Physics, Bar-Ilan University, Ramat-Gan, 52900 Israel; 2 Gonda Interdisciplinary Brain Research Center and the Goodman Faculty of Life Sciences, Bar-Ilan University, Ramat-Gan, 52900 Israel

## Abstract

Experimental evidence recently indicated that neural networks can learn in a different manner than was previously assumed, using adaptive nodes instead of adaptive links. Consequently, links to a node undergo the same adaptation, resulting in cooperative nonlinear dynamics with oscillating effective link weights. Here we show that the biological reality of stationary log-normal distribution of effective link weights in neural networks is a result of such adaptive nodes, although each effective link weight varies significantly in time. The underlying mechanism is a stochastic restoring force emerging from a spontaneous temporal ordering of spike pairs, generated by strong effective link preceding by a weak one. In addition, for feedforward adaptive node networks the number of dynamical attractors can scale exponentially with the number of links. These results are expected to advance deep learning capabilities and to open horizons to an interplay between adaptive node rules and the distribution of network link weights.

## Introduction

The brain is one of the most complex adaptive networks, where learning occurs by modifying the link weights^1^. This type of biological strategy stimulated theory and application of machine learning algorithms^2–4^ as well as recent deep learning achievements^5–8^. Accumulated experimental evidence indicate that neural network weights follow a wide distribution which is approximated by a log-normal distribution^9,10^, however, the underlying mechanism for its origination and stability is unclear^11^. Specifically, it is valuable to understand whether such a wide distribution of network weights, characterized by a small fraction of strong links, is a spontaneous outcome of a random stochastic process, or alternatively it is directed by a meaningful learning activity^12–16^.

The long-lasting assumption of learning by adaptive links was recently questioned, where experimental evidence showed that nodal adaptation occurs following its anisotropic incoming signals^17,18^, similarly to the slow learning mechanism attributed to the links^12,19,20^. Specifically, each node collects its incoming signals via several adaptive terminals (dendrites), hence all links to a terminal undergo the same adaptation, resulting in cooperative nonlinear dynamics. It presents a self-controlled mechanism to prevent divergence or vanishing of the learning parameters, as opposed to learning by links, and also supports self-oscillations of the effective learning parameters. In this paper we show that the biological reality^10,21^ of stationary log-normal distribution^9,11,22,23^ of effective link weights in neural networks is a result of such adaptive anisotropic nodes. This global distribution of the weights is a conserved quantity of the dynamics, although each effective link weight varies significantly in time. The underlying mechanism is a stochastic restoring force emerging from a spontaneous temporal ordering of spike pairs, generated by a strong nodal terminal preceding by a weak one. In addition, for feedforward adaptive node networks consisting of a few adaptive terminals, the number of dynamical attractors^24^ can scale exponentially with the number of links. These results are expected to advance deep learning capabilities^3,5,6,25,26^ where training, adaptation and generalization, information queries, occur simultaneously. It also opens horizons to find a possible universal interplay between adaptive node rules and the distribution of network link weights^26,27^.

## Results

### The model of adaptive nodes

**Figure 1:**
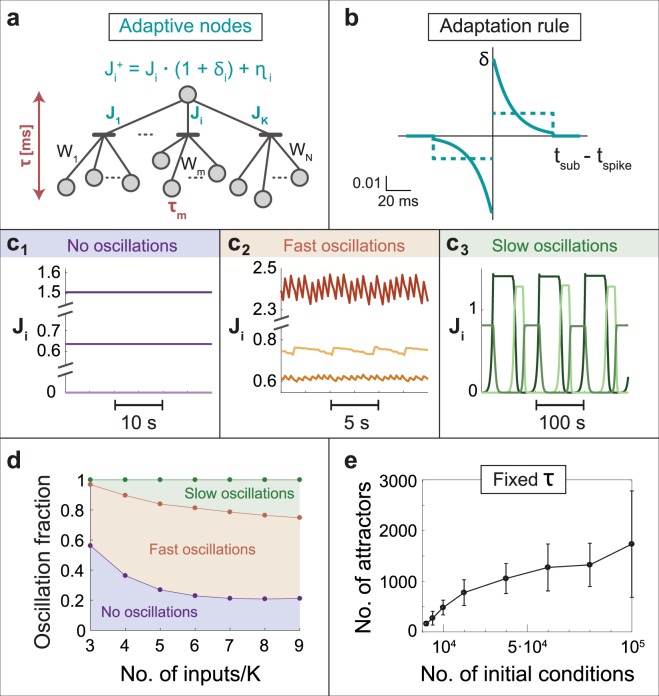
Adaptive nodes in a feedforward network. (**a**) A schema of N input units connected with fixed weights (synapses), W_m_, to K terminals (dendrites) which are connected with adaptive weights, J_i_, to an output unit. Delays between input units and the output unit are denoted by τ_m_ and the dynamics of the output unit is governed by leaky integrated-and-fire neuron, eq. (1). After an adaptation step, ${J}_{i}^{+}$ is a function of its relative change, *δ*, and an additive noise, *η*, eq. (2). (**b**) A typical profile of δ = 0.05 * exp(−|Δ|/15) * sign(Δ) (solid blue line), where Δ measures the time-lag between a sub-threshold stimulation arriving from a terminal and another one which generates a spike. Δ is measured in ms. A simplified two-level adaptive rule (dashed blue line). Both adaptive rules have a cutoff at 50 ms. (**c**) Simulation results of (**a**) with K = 3 and N = 15, where inputs are stimulated simultaneously at 5 Hz. Three types of stationary solutions for *J*_*i*_ were found: (**c**_**1**_) Fixed values. (**c**_**2**_) Fast oscillations characterized by relatively small fluctuations around a fixed value. (**c**_**3**_) Slow oscillations characterized by large fluctuations and semi-flat periods at extreme values. (**d**) Fraction of the three types of stationary solutions in (**c**), as a function of N/K for K = 3, obtained in exhaustive search simulations (Methods). (**e**) Number of different stationary firing patterns, attractors, and their standard deviation, for K = 3 and N = 9 as a function of the number of different random initial conditions for *W*_*m*_ (Methods).

In order to study the effect of nodal adaptation we modeled a node with K terminals (neuronal dendritic trees)^13^. Each terminal collects its many incoming signals via N/K time-independent link weights, W_m_, where N stands for the total number of input units (Fig. 1a). The nodal terminal is modeled as a threshold element based on a leaky integrate and fire neuron^28^

1 $$\frac{d{V}_{i}}{dt}=-\,\frac{{V}_{i}-{V}_{st}}{{\rm T}}+{J}_{i}\cdot \sum _{m=\frac{N}{k}(i-1)+1}^{\frac{N}{K}\cdot i}\,{W}_{m}\sum _{n}\,\delta (t-({t}_{m}(n)+{\tau }_{m}))$$

where V_i_(t) is the scaled voltage of the i^th^ terminal, T = 20 ms is the membrane time constant, V_st_ = 0 stands for the scaled stable (resting) membrane potential (Methods) and J_i_ stands for the i^th^ terminal weight. W_m_ and τ_m_ stand for the m^th^ link weight and delay, respectively, and the summation over n sums all input timings arriving at the m^th^ link, t_m_(n). A spike occurs when the voltage of one of the terminals crosses the threshold, V_i_ ≥ 1. After a spike is generated the terminal’s voltage is set to V_st_, and a refractory period of 2 ms occurs, where no evoked spikes are possible by any one of the terminals (Methods). Note that in order to achieve a threshold crossing, typically many inputs have to arrive to a neuron in temporal proximity via one of its terminals^29^.

For every pair of a sub-threshold stimulation via terminal i and an evoked spike from a different terminal, an adaptation step occurs for J_i_:

2 $${J}_{i}^{+}={J}_{i}\cdot (1+{\delta }_{i})+{\eta }_{i}$$

where δ_i_ and η_i_ stand for the relative change and an additive random white noise, respectively. The relative change, δ, is the same as the adaptation rule used for link weights and follows the modified Hebbian learning rule^12,19,20^ (blue-line in Fig. 1b). This relative change is a function of the time-lag between a sub-threshold stimulation and an evoked spike, t_sub_ − t_spike_, originated from a different terminal. Specifically, the relative change decays exponentially to zero for large time-lags and follows its sign (Fig. 1b). The qualitative reported results were also found to be robust to a simplified two-level adaptation rule (dashed blue-line in Figs 1b and S1).

Following recent experimental evidence, an additional ingredient is introduced, where a threshold crossing by one of the terminals now generates an evoked spike with probability^30^

3 $${{\rm{P}}}_{{\rm{spike}}}={\rm{\Delta }}t\cdot {{\rm{f}}}_{{\rm{c}}}$$

where Δt is the time-lag from the last threshold crossing, and f_c_ reflects the maximal stationary firing frequency of the neuronal terminal, e.g. 15 Hz. Note that for high stimulation frequencies (>*f*_*c*_) the nodal firing rate is saturated at *K* · *f*_*c*_, and for low stimulation frequencies (<*f*_*c*_) response failures practically vanish (Fig. 1c–e, Methods).

### Feedforward networks

When the input units are simultaneously stimulated with a common stimulation frequency, three possible types of dynamics for J_i_ are observed, according to the initial weights and delays, W_m_ and τ_m_. This is exemplified for a network with an output node with three terminals and 15 input units, without noise (K = 3, N = 15, η_i_ = 0) and 5 Hz stimulation frequency (Fig. 1c). In the first type of dynamics, all J_i_ converge to fixed values (Fig. 1c_1_). The second type is characterized by fast oscillations with relatively small fluctuations of each J_i_ around an average value (Fig. 1c_2_), and their periods are below few seconds, typically sub-seconds. The third type is characterized by slow oscillations with periods which can exceed hundreds of seconds (Fig. 1c_3_) and exists for K > 2 only^18^. They are accompanied by large variations in the amplitudes of J_i_ and consist of long plateaus at extreme values. The fraction of initial time-independent weights and delays, W_m_ and τ_m_, leading to oscillations was estimated using random sampling (Methods) for K = 3 and varies N (Fig. 1d). It increases from ~0.4 for N = 9 to ~0.8 for N = 27, indicating that the phenomenon of oscillations is a common scenario in adaptive node networks. Note that in the traditional adaptive link scenario all W_m_ converge either to zero or to above-threshold (similar to Fig. 1c_1_) and oscillations are excluded^18^.

**Figure 2:**
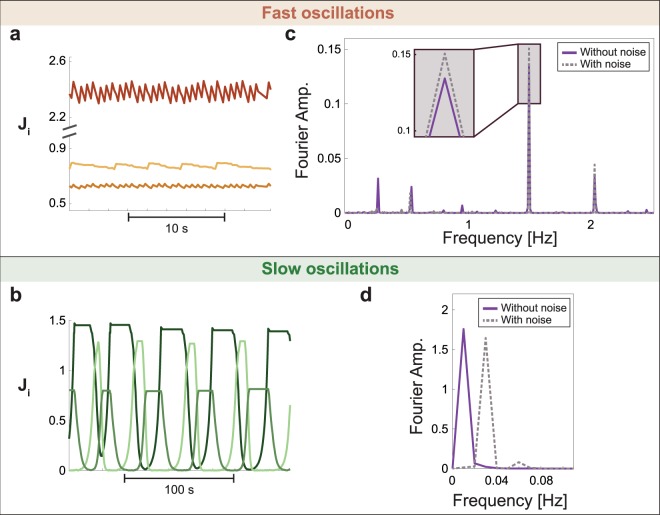
Robustness of oscillations in simulations with noise. Simulation results of an adaptive node with K = 3 and N = 15 (Fig. 1a), where inputs are stimulated simultaneously at 5 Hz. Stochastic noise, *η* is added to the adaptive step (eq. (2)) as a random number [−0.5, 0.5] · 10^−3^. (**a**) Fast oscillations characterized by relatively small fluctuations around a fixed value. (**b**) Slow oscillations characterized by large fluctuations and semi-flat periods at extreme values. (**c**) Multiplication of the Fourier components of the three adaptive weights, J_i_, taken from 100 seconds for the fast oscillations in (**a**) without noise (solid purple line) and with noise (dashed gray line). (**d**) Similar to (**c**) for the slow oscillations in (**b**) where the Fourier is done on 1000 seconds.

The robustness of the fast and slow oscillations to small stochastic noise, η in eq. (2), was examined using the Fourier analysis of the adaptive weights (Fig. 2). For fast oscillations, the noise does not affect the periods of oscillations, but only slightly affects their Fourier amplitudes (Fig. 2a,c). In contrast, for slow oscillations the noise, η, affects the periodicity which is typically shortened (Fig. 2d). This trend is a result of the noise which prevents the plateau at small values of terminal weights, J, for long periods (Fig. 2b).

The number of different stationary firing patterns, attractors, in the large N limit, can be bounded from below, for given K and delays τ_m_ (Fig. 1a). Assuming that for each terminal there are N_0_ < N/K non-zero inputs, the number of different attractors, A(N_0_), is estimated using an exhaustive random sampling for W_m_ (Methods). A lower bound for the number of dynamical attractors for the entire network with N non-zero inputs, scales as

4 $$A({N}_{0})\cdot {(\begin{array}{c}N/K\\ {N}_{0}\end{array})}^{K}$$

since for each one of the K terminals one can select a subset of N_0_ inputs among N/K, with repeated above-threshold stimulated inputs. Each one of these choices results in A(N_0_) different attractors as a result of different delays. For K = 3 and N_0_ = 3, for instance, the number of different attractors was estimated as *A*(3) ~ 1500 (Fig. 1e and Methods), indicating that eq. (4) scales as *N*^9^. For N_0_ = O(N) even with small K, e.g. K = 2, the number of different attractors is expected to scale exponentially with N. This type of input scenarios is expected in biological realizations where a neuron has only a few terminals (dendritic trees)^13^ and many thousands of links (synapses)^29^, however at each firing event only a small fraction of the input links is effectively involved^29^. Results indicate powerful computational capabilities under biological realizations with a huge number of attractors even for such a simple feedforward network with only finite number of adaptive terminals.

### Recurrent networks and log-normal distributions

**Figure 3:**
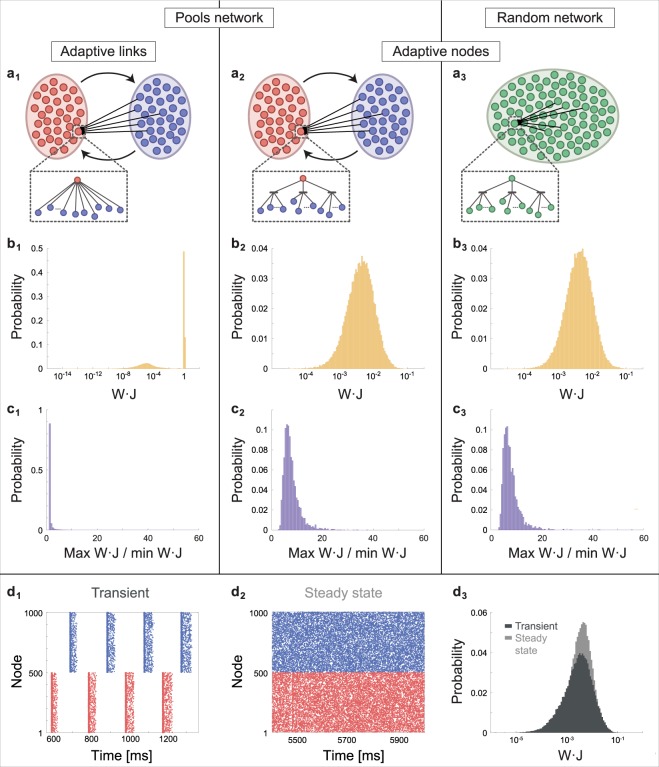
Stationary log-normal distribution of effective link weights in recurrent networks. Simulation results for three recurrent networks of size 1000 where each node has 60 non-zero inputs, *W*_*m*_, which were randomly selected from a uniform distribution [0.1, 0.2]. (**a**_**1**_) Two pools, each comprised of 500 nodes where each node is connected to 60 nodes from the other pool and with adaptive rules to link weights only, *W*_*m*_, similar to eq. (2) (Methods). (**a**_**2**_) The same network as in (**a**_**1**_), but with adaptive nodes, three terminals, K = 3. (**a**_**3**_) A random network comprised of 1000 nodes with K = 3 where each node is randomly connected to 60 nodes. (**b**) The stationary probability distribution of the effective link weights, *W* · *J*, for each network in (**a**). The effective links are presented in a log-scale, where for (**a**_**1**_) J = 1. (**c**) Probability distribution of the maximal value divided by the minimal value of the effective link weights for each network, during the last 2 seconds (Methods), indicating *W* · *J* are not frozen in (**a**_**2**_ and **a**_**3**_). (**d**_**1**_) A raster plot of the transient state in dynamics of (**a**_**2**_), indicating alternating firing between pools (color coded). (**d**_**2**_) A raster plot of the steady state of (**a**_**2**_) indicating random firing (Fig. S4). (**d**_**3**_) Probability distribution of W⋅J in log-scale for (**d**_**1**_) (dark gray) and (**d**_**2**_) (light gray), respectively, indicating that a log-normal distribution emerges already in the transient.

To this point we assumed simultaneous inputs, which is far from biological reality. In order to expand the study to non-simultaneous inputs, asynchronous stimulations are first discussed for the case of population dynamics between two pools^31^ consisting of 500 nodes each (Fig. 3a_1_ and a_2_). In the adaptive links scenario, each node receives 60 inputs from randomly selected nodes from the other pool (Fig. 3a_1_), or via three adaptive terminals for the adaptive nodes scenario (Fig. 3a_2_), using random delays from a normal distribution of 100 ms mean and a standard deviation of 2 ms. Networks are simulated using eq. (1) (Methods). For the adaptive links scenario, weights converge to biologically unrealistic limits, and are frozen at either above-threshold or practically vanish (Fig. 3b_1_ and c_1_). For the adaptive nodes scenario, the distribution of the effective weights, *W*_*m*_ · *J*_*i*_, converges to a stationary log-normal distribution (Fig. 3b_2_), however, each weight is not frozen and significantly varies along the dynamics (Figs 3c_2_ and S2). A similar stationary log-normal distribution was obtained for a random network consisting of the same 1000 adaptive nodes, where each receives inputs from 60 randomly selected nodes (Fig. 3a_3_ and b_3_). The log-normal distribution is stationary (Fig. S3), however each weight is not frozen and significantly varies along the dynamics (Fig. 3b_3_ and c_3_) such that its distribution can also be well approximated by log-normal (Fig. S2). The log-normal distribution is not attributed to the emergence of some spontaneous clustering among the adaptive nodes, as the raster plot indicates a random firing activity of each node and the entire network, without any significant structure in the Fourier spectrum (Fig. S4). For pools consisting of adaptive nodes (Fig. 3a_2_), where one of the pools was initially triggered, the raster plot initially indicates alternating firing between two synchronized pools (Fig. 3d_1_). The variation between delays results in the broadening of the firing stripes until merging occurs with random raster activity (Fig. 3d_2_), similar to the random network (Fig. 3a_2_). This broadening and merging might be a self-control mechanism to terminate an induced reverberating mode. Interestingly, the log-normal distribution for the effective weights emerges already in the transient consisting of stripes activity (Fig. 3d_3_), and only its average (and variance) are later adjusted. The firing rate of each terminal in the network (Fig. 3a_2_ and a_3_) is saturated, ~15 Hz, hence, the firing frequency of each node is ~45 Hz.

### Restoring force via spontaneous spike ordering

**Figure 4:**
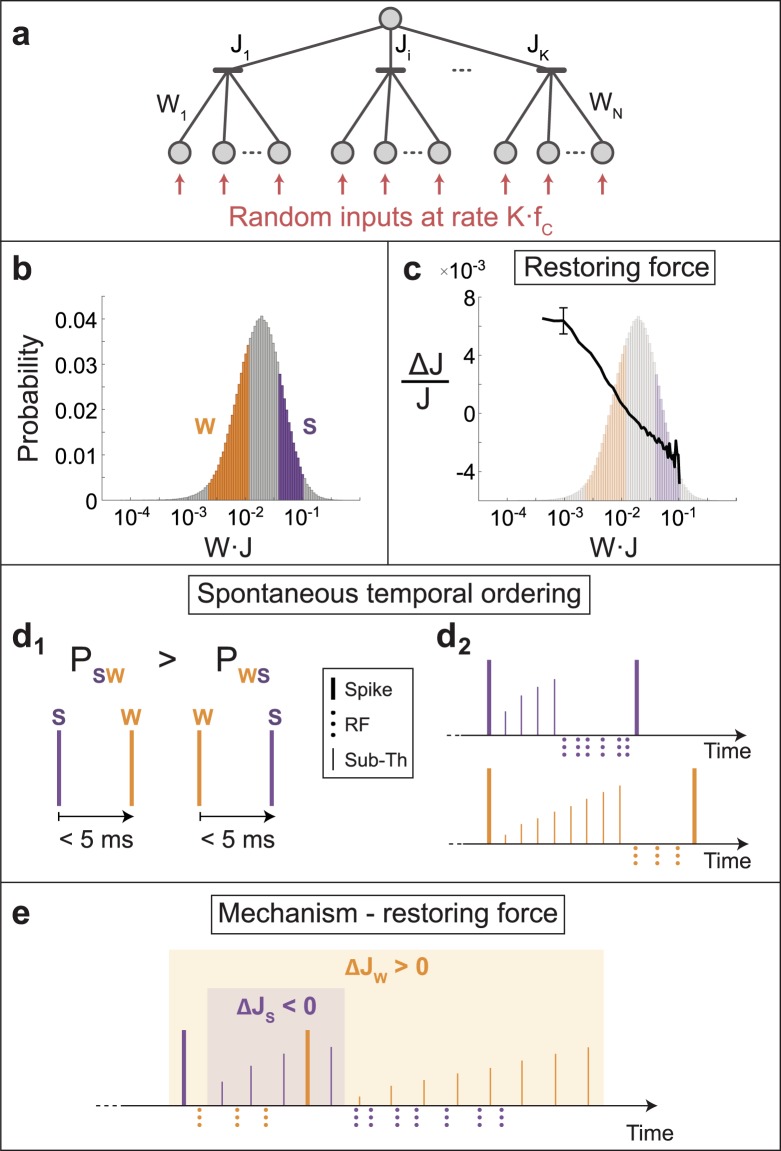
Stationary log-normal distribution emerging from spontaneous temporal ordering. (**a**) A schema similar to Fig. 1a_1_, where each input is stimulated above-threshold at random with an average rate K ⋅ f_c_. (**b**) Probability distribution of the effective link weights in log-scale for (**a**) with K = 2 and N = 120. Weak effective weights (orange) and strong ones (purple) each constitute 24% of the distribution. (**c**) The relative average change in *J* as a function of *W* · *J*, with a typical error bar (black), obtained in the stationary dynamics of (**b**) (background). Results indicate a restoring force towards approximately the most probable *W* · *J* (see also Fig. S5). (**d**_**1**_) Two scenarios and their probabilities for a pair of two spikes which occur in a time window of 5 ms. A spike originated from a strong effective weight (purple) and then a weak one (orange) occurs with probability *P*_*SW*_, and vice versa with probability *P*_*WS*_. Simulation results indicate spontaneous temporal ordering, *P*_*SW*_ > *P*_*WS*_. (**d**_**2**_) Strong and weak spikes emerge first simultaneously (left), subsequently rapid rebuilding of their membrane voltages by sub-threshold stimulations (thin lines) accompanied by response failures (RF, dotted lines) when crossing thresholds are achieved. Since the voltage of the strong spike is rebuilt faster, the next strong spike occurs before the weak one (right), explaining the emergence of the spontaneous spike ordering in (**d**_**1**_). (**e**) The most probable scenario, a weak spike consecutive a strong one ((**d**_**1**_) left), is demonstrated with accompanied sub-threshold stimulations and response failures (each background covers sub-threshold stimulations and the other color spike). Adaptation step of the strong spike is negative, Δ*J*_*S*_ < 0, since most of its sub-threshold stimulations are prior to the weak spike, whereas Δ*J*_*w*_ > 0 since all the weak sub-threshold stimulation occur after the strong spike. These stochastic opposite trends explain the mechanism for the emergence of the restoring force in (**c**).

The understanding of the underlying mechanism for the emergence of a stationary log-normal distribution requires the examination of a much simpler system imitating the network activity. We examine the dynamics of an adaptive node consisting of two terminals (K = 2) and 60 inputs, where each one of the inputs is stimulated at random and on the average at 30 Hz (Fig. 4a and Methods). The distribution of the effective weights is indeed log-normal distribution (Fig. 4b) and is practically identical to the distribution obtained in the network dynamics (Fig. 3b_3_).

**Figure 5:**
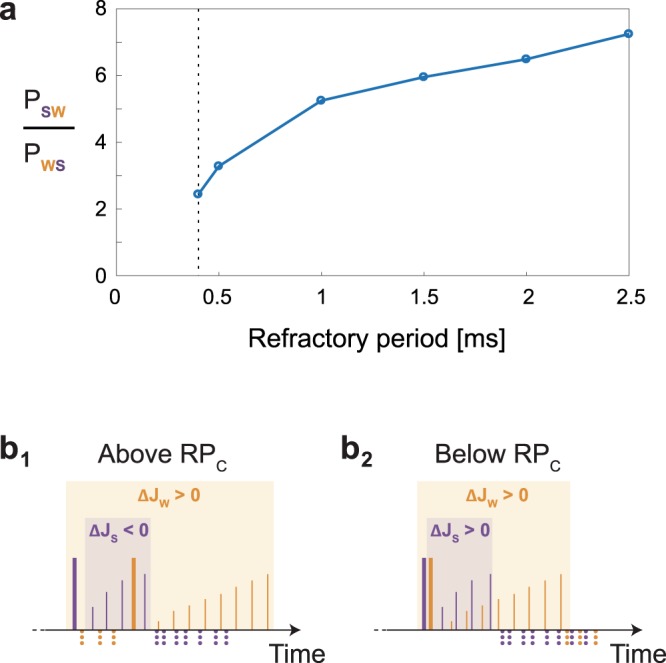
Critical refractory period (RP_C_). (**a**) The fraction P_SW_/P_WS_ as a function of the refractory period for the parameter of Fig. 4b, where P_SW_ stands for the fraction of spikes generated by J_S_ prior to a spike generated by J_W_ in a time windows of 5 ms, and P_WS_ represents the opposite scenario. Results were calculated starting from the minimal possible refractory period, 0.4 ms (black dashed line) where the log-normal distribution was obtained. (**b**) The most probable scenario, where a weak spike follows a strong one, is demonstrated with accompanied sub-threshold stimulations (solid thin lines) and response failures (dotted lines below the x-axis) for two cases. (**b**_**1**_) Above the critical refractory period (RP_C_), the time-lag between the spikes is long enough to ensure that most of the strong sub-threshold stimulations (thin purple lines) occur prior to the weak spike, resulting in a total negative adaptive step (ΔJ_S_ < 0). (**b**_**2**_) Below RP_C_, the time-lag between the spikes is too short, thus most of the strong sub-threshold stimulations (thin purple lines) occur after the weak spike, resulting in a total positive adaptive step (ΔJ_S_ > 0). Since both ΔJ_S_ and ΔJ_W_ are positive, all weights are driven to be above-threshold.

The emergence of a log-normal distribution is natural, since multiple adaptation steps of a weight, eq. (2), result in a multiplicative process, however, its stationary shape requires an explanation. The relative change in links with a given weight J, averaged over such instances during the stationary dynamics, revealed a stochastic restoring force towards the most probable J (Figs. 4c and S5). The origin of this restoring force is the emergence of spontaneous temporal ordering of pairs of spikes for a given adaptive node during the dynamical process. For simplicity we assume K = 2 and we concentrate on momentary events of the dynamics where the adaptive node has simultaneously one weak, J_W_, and one strong, J_S_, terminals, relative to the most probable values of the log-normal distribution (Fig. 4b). Next we estimate in simulations the probability of occurrence of the following two types of pairs of spikes in a bounded time window, e.g. 5 ms (Fig. 4d_1_). The first type, P_SW_, stands for a spike generated by J_S_ prior to a spike generated by J_W_, and vice versa for the second type of pairs, P_WS_. Simulation results indicate

5 $${P}_{SW} > {P}_{WS}$$

where typically *P*_*SW*_ is several times greater than *P*_*WS*_, and *P*_*SW*_ constitutes a few percent of all pairs of events (Fig. 5a). This preference, eq. (5), is exemplified using the following self-consistent argument assuming that initially the weak and the strong spikes occur almost simultaneously (Fig. 4d_2_). Since the input units of both terminals are stimulated at the same rate, the threshold crossing of *J*_*S*_ occurs before *J*_*W*_ which are both accompanied by response failures, eq. (3). Consequently, the spike generated by *J*_*S*_ occurs prior to the spike generated by *J*_*W*_ (Fig. 4d_2_). Note that the adaptation steps, eq. (2), change the two terminals from remaining strong and weak, however on the average there is a stochastic tendency for the strong spike to evoke prior the weak one.

## Discussion

The mechanism of the restoring force is a direct consequence of the spontaneous temporal ordering (Fig. 4d). A terminal that evoked a spike resets its membrane potential which rapidly increases by many sub-threshold stimulations. The threshold crossing is achieved again in several ms and is followed by many response failures. Hence, the strong terminal generates most of its sub-threshold stimulations prior to the following weak spike, whereas all the sub-threshold stimulations of the weak terminal appear after the strong spike (Fig. 4e). Following the adaptation rule, eq. (2), the strong terminal is decreased, Δ*J*_*S*_ < 0, whereas the weak terminal is enhanced, Δ*J*_*W*_ > 0 (Fig. 4e) and the restoring force is created.

A necessary ingredient in the formation of the mechanism to achieve a stationary log-normal distribution is that the majority of the sub-threshold stimulations of the strong spike occur prior to the weak one (Fig. 4e). For short refractory periods the time-lag between a pair of strong-weak spikes decreases, since the minimal time-lag between consecutive spikes decreases. Indeed, for short enough refractory periods and certainly for a vanishing one, the log-normal distribution was found in simulations to be unstable, where all effective weights are asymptotically above threshold, since both Δ*J*_*s*_ and Δ*J*_*w*_ are now positive (Fig. 5). The log-normal distribution of link weights is an emerging spontaneous feature of adaptive node networks where the essential role of the refractory period is evident. Results open the horizon to explore the possible interplay between the adaptive node rules and stationary distribution classes of the network link weights^26,27^.

## Methods

### Simulation dynamics

Each node is described by several independent terminals, and a node generates a spike when a terminal crosses a threshold (eqs. (1) and (3)). The voltage of each terminal is determined according to the leaky integrate and fire model as described in eq. (1), where T = 20 ms. For simplicity, we scale the equation such that V_th_ = 1, V_st_ = 0, consequently, V ≥ 1 is above threshold and V < 1 is below threshold. Nevertheless, results remain the same for both the scaled and unscaled equations, e.g. V_st_ = −70 mV and V_th_ = −54 mV. The initial voltage for each terminal is V_(t=0)_ = 0 and J_i_ = 1. The adaptation is done according to eq. (2), where $\delta =A\cdot \exp (-\,\frac{{\rm{\Delta }}t}{15})\cdot sign({\rm{\Delta }}t)$, and Δt stands for the time between a sub-threshold stimulation and a spike, up to a cutoff at 50 ms. The parameter *η* is chosen randomly in the range [−0.5, 0.5] · 10^−3^, and A is the adaptation step taken as 0.05, unless otherwise is stated.

### Refractory period

After a spike is generated, the terminal that evoked a spike cannot respond to other stimulations arriving in the following 2 ms. During this refractory period, all other terminals cannot evoke a spike or cross the threshold as well, but can increase their membrane potential as a result of stimulations.

### Response failure

When crossing the threshold, the terminal creates a spike with probability of Δt · f_c_, where Δt is the time-lag from the last threshold crossing by this terminal, and f_c_ reflects the maximal stationary firing frequency of the terminal. In case the terminal failed to respond its voltage is set to its previous value.

### The parameters for feedforward networks

Number of terminals = 3, number of inputs per terminal = 5, refractory period = 2 ms, link weights are randomly chosen from a uniform distribution in the range [0.1, 1.1], delays (τ) are randomly chosen from a uniform distribution in the range [1, 150] ms (Fig. 1c). Links are ordered with increasing delays, except the maximal delay which is linked to the first terminal (closing a loop). The dynamics is given by eq. (1) and is numerically solved with a time resolution of 1 ms. Initial terminal weights, J_i_, are set to 1. We assume large f_c_, hence response failures are excluded. In addition, in Fig. 1 η = 0. The robustness of the results to noise, η > 0, is demonstrated in Fig. 2. The upper bound for the terminal weights is J_i_ = 10 and the lower bound is J_i_ = 10^−6^.

### The fraction of oscillations

The fraction of each type of dynamics was estimated using 20,000 random initial conditions for the delays, τ_m_, and the weights, W_m_, (defined above) for each number of inputs per terminal (Fig. 1d).

### The number of attractors

Number of terminals = 3, number of inputs per terminal = 3. The average and the standard deviation of each point was obtained from 10–18 samples, each sample is with a fixed set of N delays (τ) and the initial conditions for the N weights are randomly sampled. In order to determine if two initial conditions lead to the same attractor, we compared the firing rate from each input link. We calculated the number of firing events for each link, and compared it with the same link from a simulation with different initial weights. If for all of the input links the difference is less than 2%, we determine that these different initial weights lead to the same attractor. For links that have low firing rates, the comparison was made between non-firing events. We obtained very similar results when the comparison was done between the firing timings for each link, instead of number of firing events (Fig. 1e).

### Recurrent network parameters

Number of terminals = 3, number of inputs per terminal = 60, f_c_ = 15 Hz, refractory period = 2 ms, adaptation step A = 0.05. The dynamics of each node is given by eq. (1) and is solved with a time resolution of 0.1 ms. Link weights are randomly chosen from a uniform distribution in the range [0.1, 0.2], delays are randomly chosen from a normal distribution with a mean of 100 ms and STD of 2 ms, initial terminal weights, J_i_, are set to 1. In order to initiate the network simulation, 0.4 of the nodes in the network are stimulated above-threshold. Spontaneous noise, external above-threshold stimulations, is randomly added with an average frequency of 0.01 Hz per node (Fig. 3).

The ratio max/min of each weight (Fig. 3c) was calculated for the last 2 seconds of the simulation, out of 50 seconds for adaptive nodes and 350 seconds for adaptive links (same running time as for Fig. 3b). For networks of adaptive links (Fig. 3c_1_) a fraction of the weights vanishes, hence the upper bound of the histogram is set to 60. The histograms (Fig. 3c_2_ and c_3_) constitute of 100 bins each. For visibility, points in the raster plots (Fig. 3d_1_ and d_2_) were 50% diluted.

### The parameters for the feedforward network with random inputs

Number of terminals = 2, number of inputs per terminal = 60, f_c_ = 15 Hz, refractory period = 2 ms, adaptation step A = 0.1, link weights are randomly chosen from a uniform distribution in the range [0.1, 0.2], initial terminal weights are set to 1 (Fig. 4). The dynamics is given by eq. (1) and is solved with a time resolution of 0.1 ms. Running time = 2500 seconds, where a transient of 200 seconds is excluded in the measurements. Strong and weak weights (Fig. 4b–e) were chosen such that 50% of the weights were between the maximum of the weak and the minimum of the strong effective weights, and in addition for each limit (maximum and minimum) 1% of the extreme weights were excluded. The force was calculated in bin size of 0.05 and defined as $\frac{({J}^{+}-J)}{\langle J\rangle }$, where 〈*J*〉 stands for the average bin value. The error bar (Fig. 4c) stands for the standard deviation of the adaptation steps belonging to each bin.

## Electronic supplementary material

Supplementary information
