# Supplementary material for: Stationary log-normal distribution of weights stems from spontaneous ordering in adaptive node networks
Source: Sci Rep. 2018 Aug 30;8:13091. doi: 10.1038/s41598-018-31523-1 (PMC6117314; doi:10.1038/s41598-018-31523-1)
Supplement: Supplementary file 1 — Supplementary information [file 41598_2018_31523_MOESM1_ESM.pdf]

Stationary log-normal distribution of weights stems from spontaneous ordering in adaptive node networks

Herut Uzan[1,+], Shira Sardi[1,+], Amir Goldental[1], Roni Vardi[1] and Ido Kanter[1,2]*

[1] Department of Physics, Bar-Ilan University, Ramat-Gan 52900, Israel

[2] Gonda Interdisciplinary Brain Research Center and the Goodman Faculty of Life Sciences, Bar-Ilan University, Ramat-Gan 52900, Israel

[+] These authors contributed equally to this work

*e-mail: ido.kanter@biu.ac.il

Supplemental Figures

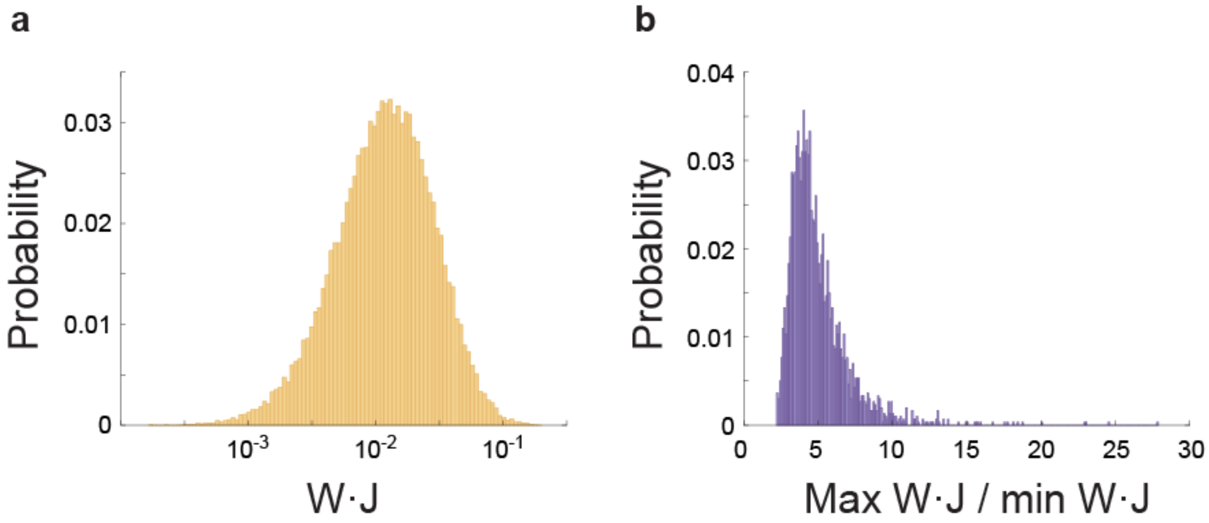

Figure S1. Robustness of the log-normal distribution to a two-level adaptation rule. (**a**) Simulation results showing the stationary probability distribution of the effective link weights, $W \cdot J$, for random network as in Fig. 3b3, but with a two level adaptive rule (dashed line in Fig. 1b). (**b**) The probability distribution of the maximal value divided by the minimal value of the effective link weights as in Fig. 3c3, using a two-level adaptation step.

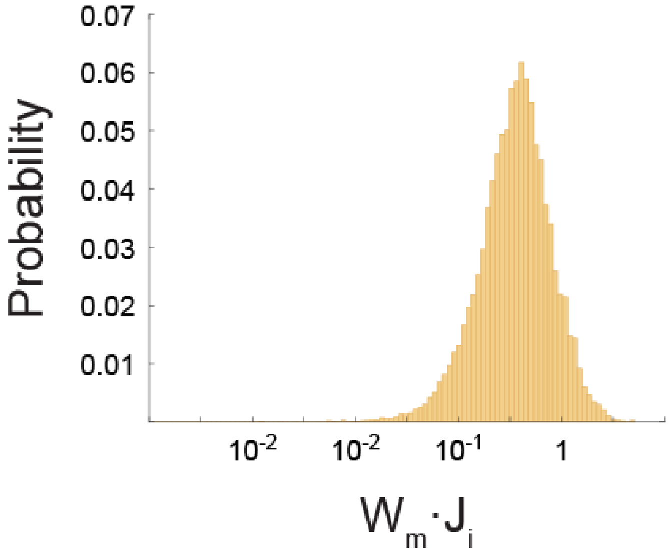

Figure S2. Time-dependent of each adaptive weight. The stationary probability distribution of one effective link weight, $W_m \cdot J_i$, indicating that each weight, J_i, is not frozen and significantly varies along the dynamics.

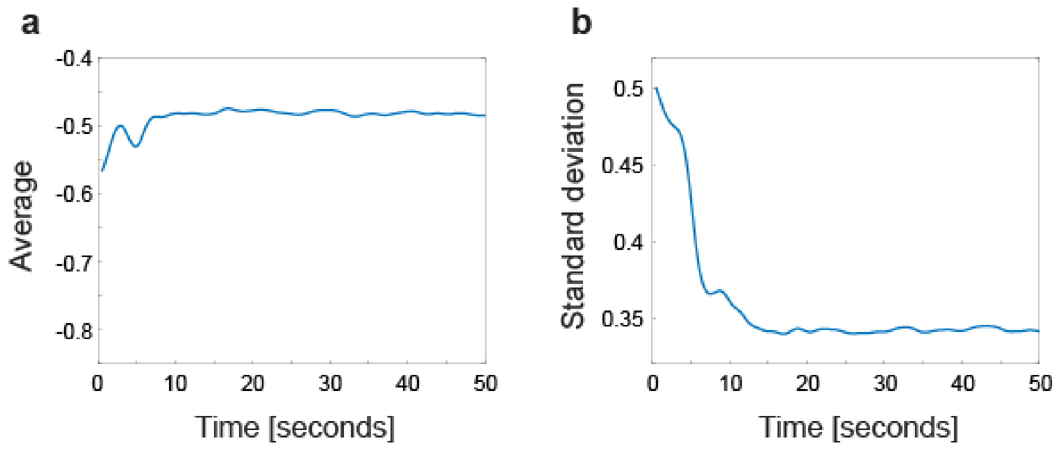

Figure S3. Stationary distribution of the log-normal distribution. The average (**a**) and standard deviation (**b**) of the log-normal distribution for the network in Fig. 3a$_2$, calculated for every second during simulations of 50 seconds. Results are smoothed with a Gaussian with window of 4 seconds, where the mean is in 2 seconds and STD = 0.8 seconds.

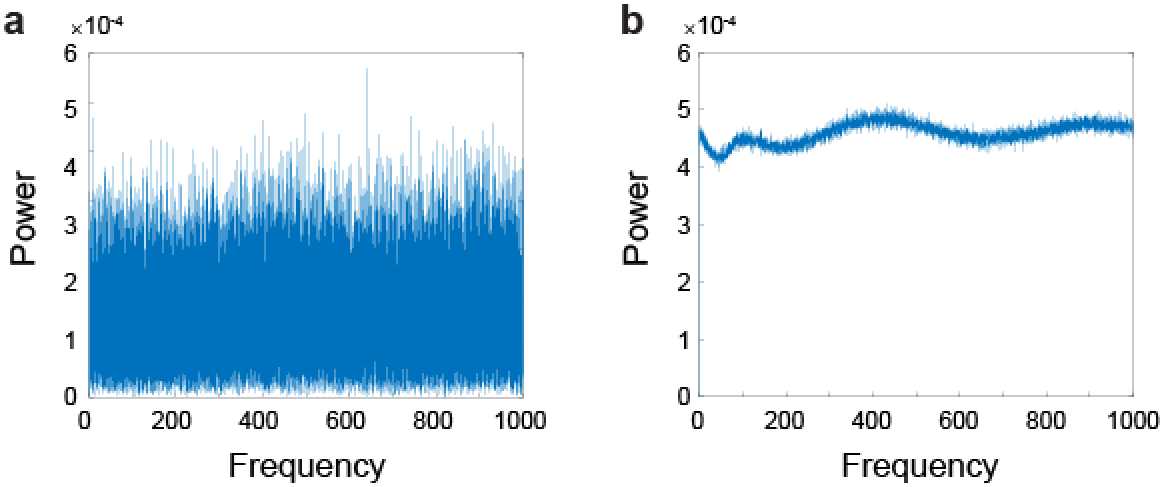

Figure S4. Fourier analysis of the network firing activity. (**a**) The Fourier analysis of the firing times of the network simulation presented in Fig. 3a_3, for a typical node. The analysis was performed on a random node using fast Fourier transform after subtracting its mean. (**b**) The normalized sum of the Fourier analysis of each node of the network (parameters as in Fig. 3a_3). Results indicate a random firing activity of each node and the entire network, without any significant structure, up to 1000 Hz.

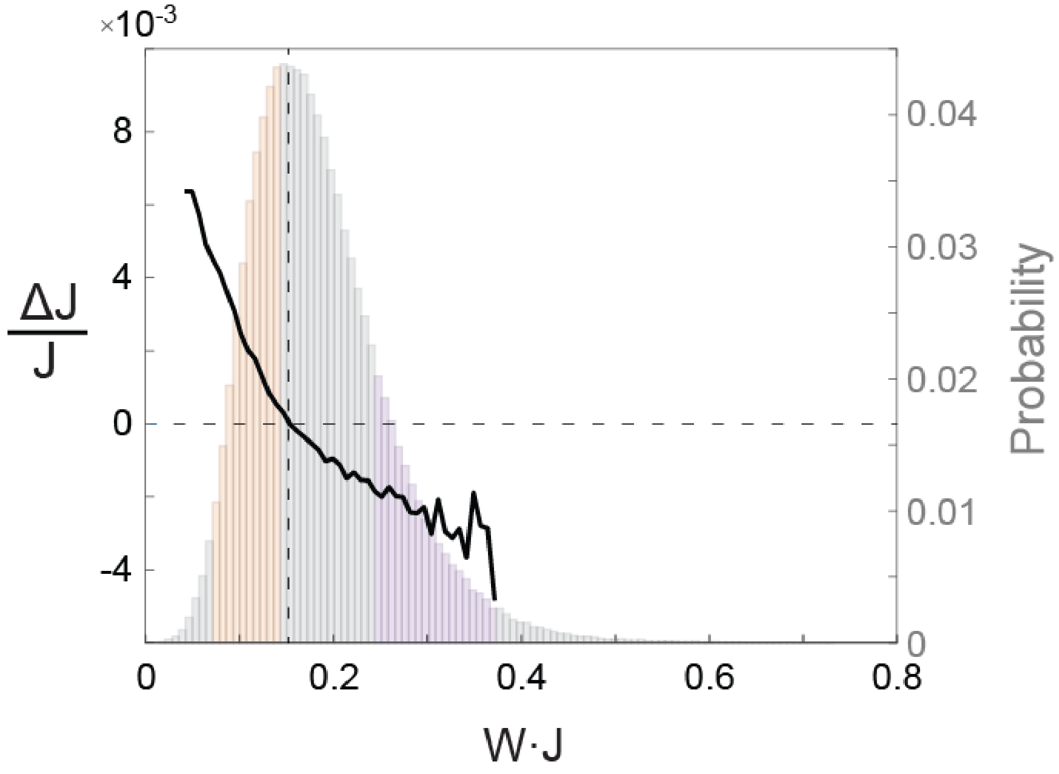

Figure S5. The sign change of the restoring force. The relative average change in J as a function of $W \cdot J$ (black), obtained in the stationary dynamics of Fig. 4c. The distribution of the effective weights is presented in the background, strong and weak effective weights are denoted in purple and orange, respectively (as in Fig. 4c). Results indicate a restoring force towards approximately the most probable $W \cdot J$.
